# The aftermath of boxing revisited: identifying chronic traumatic encephalopathy pathology in the original Corsellis boxer series

**DOI:** 10.1007/s00401-018-1926-8

**Published:** 2018-10-30

**Authors:** Marc H. Goldfinger, Helen Ling, Bension S. Tilley, Alan K. L. Liu, Karen Davey, Janice L. Holton, Tamas Revesz, Steve M. Gentleman

**Affiliations:** 1 Division of Brain Sciences, Department of Medicine, Imperial College London, 4th Floor Burlington Danes Building, Hammersmith Campus, Du Cane Road, London, W12 0NN UK; 2 Queen Square Brain Bank for Neurological Disorders, UCL Institute of Neurology, University College London, London, UK; 3 Reta Lila Weston Institute for Neurological Studies, UCL Institute of Neurology, London, UK; 4 Department of Molecular Neuroscience, UCL Institute of Neurology, University College London, London, UK

In 1973, Corsellis and colleagues presented their findings of neuropathological changes in retired boxers in their seminal paper entitled ‘The Aftermath of Boxing’ [2]. The neuropathological changes associated with boxing, such as ventricular dilatation, cavum septum pellucidum and neurofibrillary tangle pathology highlighted by the authors would go on to set the groundwork for our understanding of chronic traumatic encephalopathy (CTE) [2–4, 9, 10]. In 2016, under the guidance of NINDS/NIBIB, a consensus was reached amongst neuropathologists with expertise in neurodegenerative brain pathologies on the preliminary neuropathological diagnostic criteria for CTE, with a pathognomonic lesion of hyperphosphorylated tau in both neurons and astrocytes around small blood vessels at the base of cortical sulci [8]. Here, we sought to audit the neuropathological diagnosis of the boxers in the Corsellis series using the proposed preliminary criteria for CTE. Employing modern immunohistochemical techniques to tissue sections available from 14 of the original 15 cases, we screened for the deposition of hyperphosphorylated tau. The archival brain tissue from the Corsellis collection has been in fixative since the early 70s and sampled by many researchers over the past 45 years. This has limited the scope of our study and may affect our results in that we did not have access to all recommended regions outlined in the consensus criteria [8]. We therefore sampled multiple sites within the frontal and temporal cortices and systematically examined serial sections from these regions. The available brain regions and immunohistochemical staining applied are summarised in the supplementary tables. Antigen retrieval was performed to optimise staining in tissue fixed for prolonged periods.

Our findings indicate that seven of the 14 cases (mean age 69) fulfilled current CTE diagnostic criteria (Fig. 1). One of the seven CTE cases was found to have concomitant Alzheimer’s pathology, whilst the remaining six had CTE as the sole diagnosis. In the remaining cases, mixed pathologies were observed (see supplementary information for details). The presence of ARTAG pathology was identified in ten cases, including all seven CTE cases. Two cases were found to have no neuropathology (mean age 74.5) and two cases were found to have only ARTAG pathology (mean age 64). Further information on demographics, clinical data and boxing history of these subjects can be found in the supplementary data. It is important to note that we were unable to verify any boxing history for the two cases with no neuropathological abnormality.

**Fig. 1:**
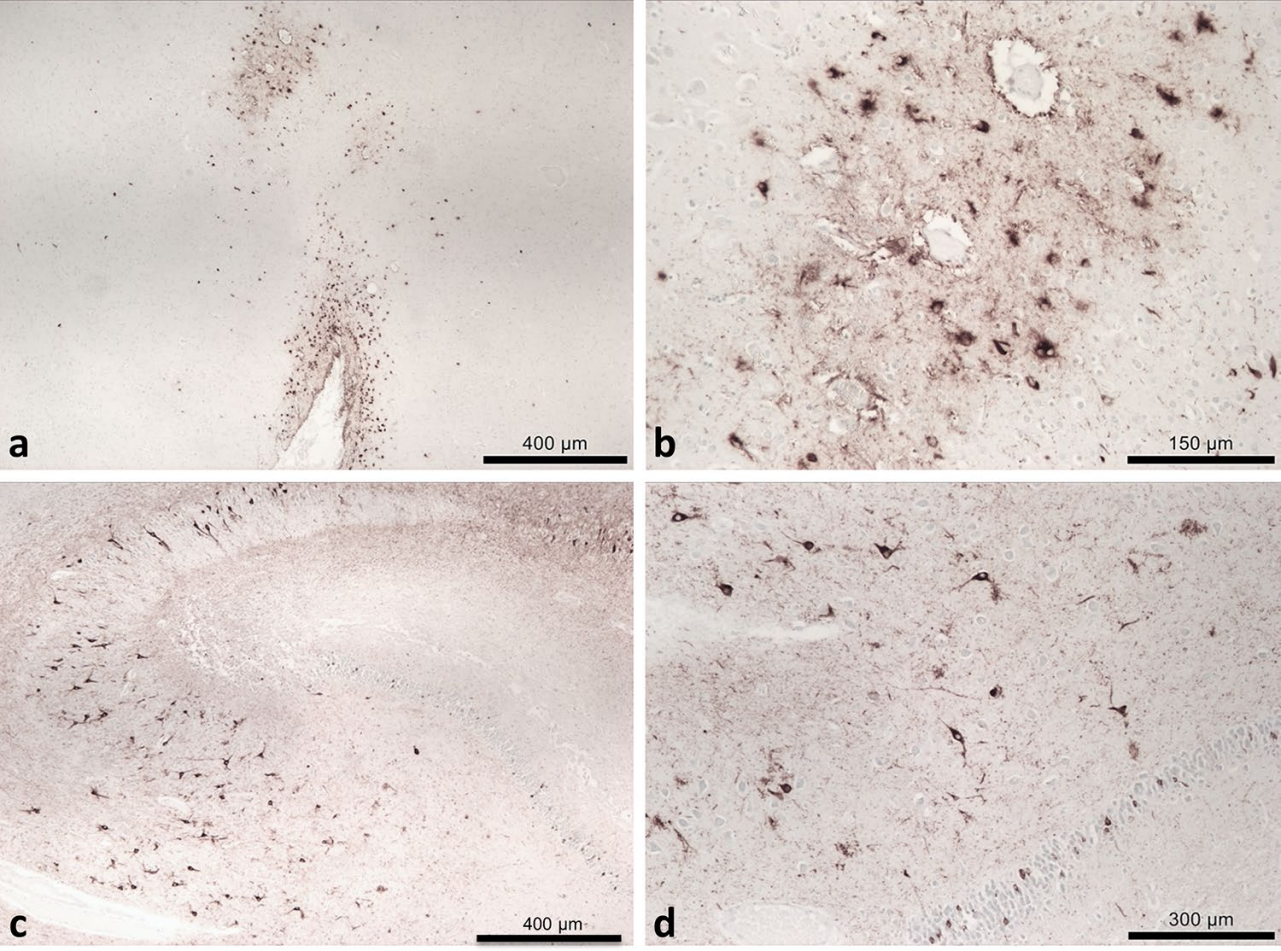
Tau (AT8) immunohistochemistry. **a** Pathognomonic tau lesions of CTE in case 7, a 62-year-old male, in the frontal cortex, with sulcal depth neuronal and astrocytic inclusions with a higher magnification (**b**) of the perivascular tau in the base of the affected sulcus. **c** CA4 region of the hippocampus in case 7, showing neurofibrillary tangles and proximal dendritic swellings, an example of tau supportive features. **d** A higher magnification of tau pathology in the dentate gyrus and CA4 of the hippocampus in case 7, another example of supportive tau features

The presence of additional tau pathologies is an interesting finding. A recent meta-analysis of 32 studies by Li and colleagues [6] demonstrated that repetitive head impact (RHI) significantly increases the relative risk of developing dementia and AD; however, recent publications indicate that TBI may not increase the risk of AD [12]. To further complicate the issue, TBI has also been shown to increase the risk for the onset other proteinopathies such as Lewy Body dementia [1], whilst the presence of multiple proteinopathies in individuals has been associated with advanced age [11]. ARTAG is a newly recognised histological entity with tau accumulation restricted to the glia rather than neurons, commonly observed in older individuals [5, 7]. The overlapping histological features of ARTAG and CTE and the frequent concomitant findings of their features in the same individual raise the question of these two histological entities being on a spectrum of the same disorder.

Our results indicate that not all individuals exposed to RHI from boxing or other high impact sports inevitably develop CTE pathology. RHI may not be sufficient on its own to lead to CTE. It is possible that environmental and genetic risk or protective factors may also play a role in the onset of CTE pathology.

## Electronic supplementary material

Below is the link to the electronic supplementary material.

Supplementary material 1 (DOCX 5160 kb)
