# Supplementary material for: The aftermath of boxing revisited: identifying chronic traumatic encephalopathy pathology in the original Corsellis boxer series
Source: Acta Neuropathol. 2018 Oct 30;136(6):973–4. doi: 10.1007/s00401-018-1926-8 (PMC6280830; doi:10.1007/s00401-018-1926-8)
Supplement: Supplementary file 1 — Supplementary material 1 (DOCX 5160 kb) [file 401_2018_1926_MOESM1_ESM.docx]

| **Case no.** | **Tau pathologies**  **(Mandatory features)** | **Tau pathologies**  **(Supportive features)** | | | | | **Non-tau pathologies (Supportive features)** | | **ARTAG with thorn-shaped astrocytes**  **(Non-diagnostic and non-supportive features)** | | | **Diagnosis** | | **Demographics** | |
| --- | --- | --- | --- | --- | --- | --- | --- | --- | --- | --- | --- | --- | --- | --- | --- |
|  | **Perivascular, depths of cortical sulci, irregular pattern (neuronal & astrocytic)** | **Superficial cortical layers** | **CA2 (NFTs, Pre-Ts, GTs), CA4 (proximal dendritic swellings)** | **Subcortical nuclei (neuronal & astrocytic)** | **Subpial & periventricular thorny astrocytes** | **Large grain-like & dot-like structures** | **Dilatation of III^rd^ ventricle, septal abnormalities** | **TDP-43** | **Subcortical white matter (patchy)** | **Mediobasal regions (subependymal, periventricular, perivascular)** | **Amygdala or hippocampus** | **Original clinical diagnosis** | **Revised neuropathological diagnosis** | **Age at death** | **Duration of boxing (years)** |
| **1** | Present (F) | Present (F,T) | NA | Present | Present | - | Present | Present | Present | Present | NA | Punch drunk | CTE | 63 | 14 |
| **2** | Present (F) | Present (F) | NA | Present | Present | - | Present | - | Present | - | NA | Punch drunk | CTE | 77 | 19 |
| **3** | Present (F) | Present (F,T) | NA | Present | Present | - | Present | Present | - | Present | NA | Punch drunk | CTE | 62 | 10 |
| **4** | - | Present (F) | NA | Present | Present | - | Present | - | Present | Present | NA | Punch drunk | Progressive supranuclear palsy/ARTAG | 68 | 25 |
| **6** | Present (F) | Present (T) | - | Present | Present | Present | Present | - | Present | Present | NA | Dementia | CTE | 83 | 25 |
| **7** | Present (F) | Present (F) | CA4 | Present | Present | - | Present | - | - | Present | - | Pre-senile dementia | CTE | 62 | 19 |
| **8** | Present (T) | Present (F) | CA2,CA4 | Present | Present | - | Present | - | Present | Present | - | Punch drunk  TIA | CTE | 71 | 23 |
| **9** | - | - | CA4 | Present | Present | - | Present | - | Present | Present | Present | Punch drunk,  pre-senile dementia & parkinsonism | ARTAG/Lewy body dementia | 72 | 12 |
| **10** | Present (F) | **NA | CA4, CA2 | Present | Present | **NA | NA | - | - | Present | - | Punch drunk | CTE/Alzheimer’s disease | 67 | NK |
| **11** | - | Present | NA | Present | Present | - | - | - | - | Present | Present | Neurosyphilis (normal CSF serology post-treatment), otherwise active & well | ARTAG | 67 | NK |
| **12** | - | - | NA | - | - | - | Present | - | - | - | NA | Registered blind, otherwise active & well | No diagnosis | 91 | NK |
| **13** | - | **NA | NA | - | - | **NA | Present | - | - | - | NA | Punch drunk | Alzheimer’s disease | 56 | 30 |
| **14** | - | - | - | Present | Present | - | Present | - | - | Present | NA | Healthy until fatal subarachnoid haemorrhage | ARTAG | 61 | 28 |
| **15** | - | - | NA | - | - | - | Present | - | - | - | - | Healthy until fatal RTA | No Diagnosis | 58 | NK |

Table 1. CTE diagnostic criteria, including diagnosis and demographics of all 14 cases

-: Absent, *NA: not applicable or cannot comment in view of missing tissue, **NA: not applicable or cannot comment in view of severe Alzheimer’s disease pathology, ARTAG: age-related tau astrogliopathy, CA2 & CA4: CA2 & CA4 hippocampal subregions, NFTs: Neurofibrillary tangles, Pre-Ts: pretangles, GTs; Glial tangles, F: frontal cortex, P: parietal cortex, T: temporal cortex, TDP-43: Transactive response DNA-binding protein 43 kDa pathology in hippocampus, CSF: Cerebral spial fluid, TIA: Transient ischaemic attack, RTA: Road traffic accident

Table 2. Summary of all sampled regions in the 14 cases.

| **Case Number** | **Frontal Cortex** | **Cingulate**  **Cortex** | **Nucleus Accumbens** | **Temporal**  **Cortex** | **Basal Forebrain** | **Hypothalamus** | **Amygdala** | **Thalamus** | **Anterior Hippocampus** | **Posterior Hippocampus** | **Occipital Cortex** | **Parietal Cortex** | **Cerebellum** | **Pons** | **Medulla** |
| --- | --- | --- | --- | --- | --- | --- | --- | --- | --- | --- | --- | --- | --- | --- | --- |
| **1** | X |  |  | X | X |  |  |  |  |  | X |  | X | X | X |
| **2** | X | X | X | X | X |  |  |  |  |  | X |  | X | X | X |
| **3** | X | X | X | X | X | X | X |  |  |  |  | X | X | X | X |
| **4** | X | X | X | X |  |  |  |  |  |  | X |  | X |  |  |
| **6** | X | X | X | X | X | X |  |  |  |  | X |  | X | X | X |
| **7** | X |  | X | X | X |  |  |  |  | X | X |  | X | X |  |
| **8** | X | X | X | X |  |  |  |  |  |  | X |  | X | X | X |
| **9** | X |  | X | X | X |  | X | X |  | X | X |  | X |  |  |
| **10** | X |  | X | X |  |  |  |  | X | X | X |  | X | X | X |
| **11** | X |  | X | X |  | X | X |  | X |  | X |  | X | X | X |
| **12** | X | X | X |  |  |  |  |  |  |  | X |  |  |  | X |
| **13** | X | X | X | X |  |  |  |  |  |  |  |  | X |  | X |
| **14** | X |  | X | X |  | X |  |  |  | X |  |  | X | X | X |
| **15** | X |  | X | X |  |  |  |  |  |  | X |  | X | X | X |

X: Regions sampled for this study.

**Materials and methods**

*Archival tissue*

Formalin-fixed brain tissue from 14 of the original 15 boxers was obtained from the Corsellis collection, an archival collection run by the West London Mental Health Trust (http://www.wlmht.nhs.uk/). The brain tissue material of one case (Case 5) was not available. Ethical approval was granted through BRAIN UK Ref 15/019, by the South Central- Hampshire B ethics committee. Areas for screening were chosen based on tissue availability and the NINDS/NIBIB consensus criteria ([McKee, Cairns, et al., 2016](#_ENREF_18)). These regions always included middle frontal gyrus and superior temporal gyrus. Other regions sampled based on NINDS/NIBIB criteria included the hippocampus, entorhinal cortex, amygdala, thalamus, basal ganglia with nucleus basalis of Meynert (nbM), midbrain including substantia nigra, pons including locus coeruleus, medulla including dorsal motor nucleus of the vagus, cerebellum and dentate nucleus. Where available, the temporal pole and superior frontal gyrus were also included as recommended by NINDS/NIBIB criteria for extra sampling for high suspicion of CTE pathology (Table 1). Macroscopic descriptions of the tissue, including ventricular dilatation and nigral pallor, were taken from the original manuscript because it was not possible to assess these features form the tissue available. Original case numbers corresponding to those included in the original Corsellis manuscript were used for this study([Corsellis et al., 1973](#_ENREF_2)).

*Immunohistochemical process*

Tissue sections were dewaxed and rehydrated as described above. Then, sections were immersed in 1% hydrogen peroxide (H_2_O_2_)/PBS (pH 7.4) for 30 minutes at room temperature (RT) to quench endogenous peroxidase activity. Following this, sections were washed in distilled water for 5 minutes and antigen retrieval procedures were performed (if necessary) as described in table XX. Next, sections were rinsed in distilled water (5 minutes) and PBS (3 x 5 minutes) before incubation with primary antibodies at 4˚C overnight in a humidity chamber. Concentration used for various antibodies are listed in table X and the diluent was 0.3% TritonX-100 in PBS.

On the second day, tissue sections were washed with PBS (3 x 5 minutes; RT) and signal amplification techniques were carried out. In the case of rabbit or mouse raised antibodies, either the Super Sensitive Polymer-HRP immunohistochemistry detection system (Biogenex, US, QD410-YAXE) or ImmPRESS™ Excel Amplified HRP Polymer Staining Kit (Vector Labs, UK, MP7601) was used. As per the the Super Sensitive Polymer-HRP immunohistochemistry detection system protocol, tissue sections were incubated in the Super Enhancer reagent for 20 minutes (RT). Following a brief wash in PBS (2 x 5 minutes), they were incubated with a tertiary layer complex consisting of polymer-HRP for 30 minutes at RT. Next, tissues were washed in PBS (3 x 5 minutes) and visualised with DAB from the kit (10 minutes at RT).

Following staining, sections were washed in distilled water (2 x 5 minutes), briefly counterstained with Mayer’s haematoxylin (approximately 1 minute) and rinsed in running tap water. Sections were then dehydrated and cleared as described above and coverslipped with DPX.

*Pre-treatment and antigen retrieval*

All pre-treatments were performed following peroxidase treatment of the tissue in the immunohistochemistry procedure.

*Steaming*

The steamer (Argos, XX) was filled with distilled water and turned on and buffer (citric acid buffer, pH 6.0 or EDTA, pH 8.0) was placed in the steaming bowl to preheat. The buffer solution was heated for a minimum of 15 minutes prior to use, until the desired temperature of 96° C was reached. Samples were placed in the buffer and steamed for 20 minutes in total. Following steaming, the container of buffer was removed from the steamer and placed in ice to allow to cool gradually. Once at room temperature (RT), the slides were then submerged in distilled water to continue with staining.

*Pressure Cooking*

The pressure cooker (MEDIXX) was filled to the allotted line with distilled water. A container with buffer (citric acid buffer, pH 6.0 or EDTA, pH 8.0) was added and the samples placed inside. The pre-programmed cycle of 35 minutes was turned on and the pressure cooker reached a temperature pf 115°C. Following pressure cooking, the container of buffer was removed from the steamer and placed in ice to allow to cool gradually. Once at RT, the slides were then submerged in distilled water to continue with staining.

*Formic Acid treatment*

A solution of 80% Formic Acid (in distilled water) and leave the solution for 15 minutes at RT. Slides were subsequently washed in distilled water.

*Neuropathological diagnosis of CTE*

For the neuropathological diagnosis of CTE, the diagnostic criteria from the first NINDS/NIBIB consensus meeting (McKee et al., 2016) were applied to all cases. Cortical sections were examined for pathognomonic lesions which can be defined as an accumulation of hyperphosphorylated tau in both neurons and astrocytes, distributed around small blood vessels at the depths of cortical sulci and in an irregular pattern. CTE supportive features included NFTs and pre-tangles affecting the superficial layers of the cortex (laminae II and III); pre-tangles or NFTs in CA2 subregion and pre-tangles and proximal dendritic swellings in CA4 subregion of the hippocampus; neuronal and astrocytic tau in subcortical nuclei; thorn shaped astrocytes at the subpial glia limitans and periventricular regions; and large grain-like and dot-like structures. Consensus agreement was made between three of the investigators TR, JLH and SG, once the primary assessment by MHG had been carried out.

Table 3. Primary antibodies used in the study

| **Antigen** | **Catalogue No** | **Company** | **Host** | **Clonality** | **Dilution** | **Pre-treatment** |
| --- | --- | --- | --- | --- | --- | --- |
| Phospho-Tau (AT8) | MN1020 | Thermo Scientific | Mouse | Monoclonal | 1:1600 | None |
| Amyloid-β (4G8) | SIG-39220 | Biolegends | Mouse | Monoclonal | 1:15000 | Formic Acid  (80% for 15 minutes) |
| a-synuclein (α-syn, 42) | 610787 | Becton Dickson (BD) | Mouse | Monoclonal | 1:4000 | Formic Acid  (80% for 15 minutes) |
| TDP-43 | ab57105 | Abcam | Mouse | Monoclonal | 1:2000 | Steam  20 minutes in citrate buffer |

Table 4. Staining protocol for available brain regions

| **Region** | **H&E** | **AT8** | **Aβ** | **αSYN** | **TDP43** |
| --- | --- | --- | --- | --- | --- |
| Frontal cortex | X | X | X | X | X |
| Temporal cortex | X | X | X | X | X |
| Parietal cortex | X | X | X | X | - |
| Hippocampus & entorhinal cortex | X | X | X | X | X |
| Amygdala | X | X | X | X | X |
| Basal ganglia and internal capsule | X | X | X | X | - |
| Pons including locus coeruleus | X | X | X | X | - |
| Midbrain including Substantia Nigra | - | - | - | - | - |
| Medulla including dorsal motor nucleus of vagus | X | X | X | X | - |
| Cerebellar cortex and dentate nucleus | X | X | X | X | - |

Shaded area represents the recommended regions for sampling by the preliminary NINDS criteria for the neuropathological diagnosis of CTE. X: Sampled, -:not Sampled

Table 5. Demographics and clinical data.

| **Case No.** | **Age at symptom onset** | **Disease duration (years)** | **Family history** | **Presenting symptoms & disease progression** | **Progressive dementia**+ | **Behavioural changes** | **Mood changes** | **Motor impairment** | **Investigations** | **Cause of death** | **Age at death** | **Final clinical Diagnosis** |
| --- | --- | --- | --- | --- | --- | --- | --- | --- | --- | --- | --- | --- |
| Case 1* | 25 | 38 | - | Dramatic personality changes towards end of his successful boxing career in his mid-20’s, excessive alcohol consumption, gambled heavily. In his 30’s, episodic memory impairment, violent outbursts, frontal disinhibition, self-neglect. Advanced dementia, dysarthria, unsteady gait in last 3 years. | Yes  (Memory impairment in his 30’s) | Lifestyle indulgences, excessive alcohol intake, gambled heavily, reckless generosity, social inappropriateness, frontal disinhibition, ‘disgusting behaviour’, multiple RTAs, social isolation, lived in destitute. | Violent outbursts (30’s) | Unsteady gait in his 60’s, ‘could hardly walk’, dysarthria. Extensor plantar response. | - | Bronchopneumonia  Metastatic prostate carcinoma | 63 | Punch drunk |
| Case 2*^ | 55 | 22 | - | Childlike behavioural change, followed by memory impairment in late 50’s, disorientation, then cerebellar signs and hand tremor aged 67, headache, and advanced dementia aged 71 | Yes  (behavioural change followed by memory impairment, intermittent disorientation to place, lacked insight. In late 60’s, incontinence) | Childish (aged 55) | Euphoria (50’s) | Cerebellar signs with gaze-evoked nystagmus, dysarthria, dysmetria, dysdiadochokinesia, unable to tandem walk, wide-based gait (50’s), hand tremor (60’s) | Air encephalogram showed dilated ventricles with cortical atrophy, and CSP.  EEG showed moderate bilateral abnormality with intermittent delta waves in pre-central regions | Bronchopneumonia  Metastatic prostate carcinoma | 77 | Punch drunk |
| Case 3* | 36 | 27 | - | Impairment of episodic memory, ‘get muddled’, paranoid delusions, irritability, aggression, frontal features, followed by dysarthria, parkinsonism, gait impairment and falls. | Yes  (progressive memory impairment, confusion, ‘head never felt clear’) | Paranoid delusions (theft, infidelity), hypersexuality, physical violence, aggression, rage, hoarding, swearing | Irritability | Parkinsonism (drooling, hypomimia, dysarthria, rigidity, shuffling gait, pill-rolling tremor, marked bradykinesia; aged 44), ataxia with dysmetria, dysdiadochokinesia (aged 56), pseudobulbar palsy, brisk jaw jerk, wasting of small hand muscles, fasciculation in triceps, deltoid. | Air encephalogram showed dilated ventricles with cerebral atrophy and CSP. | Bronchopneumonia | 62 | Punch drunk |
| Case 4 | 64 | 5 | - | Progressive unsteadiness, dysarthria, mild cognitive impairment, followed by hypomimia and leg tremor (aged 64).  Background of dysarthria, hand tremor, spastic wide-based gait from mid-30’s without deterioration. | Yes  (Mild cognitive impairment with disorientation in time and place) | Aggression | Irritability | Hypomimia, tremor & rigidity of limbs, shuffling gait (aged 69) | Air encephalogram showed dilated ventricles with cerebral atrophy. | Bronchopneumonia | 68 | Punch drunk |
| Case 6* | 32 | 51 | - | Gait impairment, followed by dysarthria, rage and aggression, violence since his 30’s, progressive dementia, paranoid in his 60’s, and parkinsonism in last 3 years of life | Yes  (significant dementia with predominant frontal behavioural changes in his late 60’s, incontinence in his 80’s) | Aggressive, rage, violent, hypersexuality, impulsivity, explosivity, paranoid delusions | Emotional lability | Hypomimia, slow shuffling gait in his 80’s | - | Bronchopneumonia Progressive dementia | 83 | Dementia |
| Case 7* | 50 | 12 | - | Impairment of episodic memory, frontal lobe features, aggressive behaviours, severe headache | Yes  (progressive memory impairment and executive dysfunction with predominant frontal features. Aged 59, advanced dementia) | Disinhibition, hypersexuality, aggressive, excessive alcohol consumption | Irritability, mood swing, apathy | Ataxia and cerebellar signs (aged 54) | Air encephalogram showed dilated ventricles with cortical atrophy, basilar arteriosclerosis.  EEG showed abnormal excess of bursts of delta waves. | Bronchopneumonia Progressive dementia | 62 | Pre-senile dementia |
| Case 8* | 60 | 11 | NK | Repeated episodes of LOC followed by transient ataxia, left hemiparesis, persistent action hand tremor, then childish behavior, memory impairment, disinhibition, violence | Yes  (memory impairment in last few years) | Childish, disinhibition (go into the street unclothed), irrational acts, physical violence | - | Ataxia (aged 60) and tendency to fall, tremor (60’s) | - | Myocardial infarction | 71 | Punch drunk  TIA |
| Case 9 | 61 | 11 | - | Memory impairment, violent outburst, followed by asymmetrical parkinsonism | Yes  (since early 60’s, diagnosed with dementia aged 66, and spent last 7 years in a psychiatric hospital) | Violent outbursts | Suicide attempt (aged 65) | Asymmetrical parkinsonism with left rest hand tremor, tongue tremor, cogwheel rigidity and hypomimia | - | Bronchopneumonia | 72 | Punch drunk,  pre-senile dementia & parkinsonism |
| Case 10* | 40 | 27 | NK | Memory impairment | Yes  (since aged 60, marked loss of recent memory, disorientated, spent last 4 years in a psychiatric hospital) | Paranoid delusion (aged 60),  Always known as a violent man | - | - | - | Progressive dementia | 67 | Punch drunk |
| Case 11 | NA | NA | NK | (Asymptomatic) | - | - | - | - | EEG showed slowing the cerebral activity.  Left carotid angiogram showed features of cerebral atrophy | Congestive heart failure | 67 | Neurosyphilis (normal CSF serology post-treatment), otherwise active & well |
| Case 12 | NA | NA | NK | (Asymptomatic) | - | - | - | - | - | Myocardial infarction | 91 | Registered blind, otherwise active & well |
| Case 13 | 49 | 7 | - | Cognitive impairment with slowing of speech and gait, difficulty operating machinery at work, followed by neglect of personal appearance, aggressive behavior, wandering, disorientation and dysphasia. Aged 54, no speech, did not recognize his family, no speech output, bedridden. | Yes  (executive dysfunction, dressing dyspraxia, dysphasia, memory impairment) | Paranoid, aggressive, violent | Emotional lability | Generalized slowing & gait impairment (aged 49) | Air encephalogram showed dilated ventricles with cerebral atrophy.  EEG showed dysrhythmia. | Bronchopneumonia | 56 | Punch drunk |
| Case 14 | NA | NA | - | (Asymptomatic) | - | - | - | - | - | Subarachnoid haemorrhage | 61 | Healthy until fatal subarachnoid haemorrhage |
| Case 15 | NA | NA | - | (Asymptomatic) | - | - | - | - | - | Cerebral haemorrhage from RTA | 58 | Healthy until fatal RTA |

*: Cases with path-confirmed CTE applying the most recent NINDs criteria ([McKee, Cairns, et al., 2016](#_ENREF_17))

**^**This case (Case 2 in the Corsellis’ series ([Corsellis et al., 1973](#_ENREF_2))) corresponds to Case 3 reported in another historical boxer series ([Spillane, 1962](#_ENREF_29))

-: Absent, LOC: Loss of consciousness, NA: Not applicable, NK: Not known, RAF: Royal Air Force, RHI: Repetitive head impacts, RTA: Road traffic accidents, CSP: Cavum septum pellucidum, EEG: Electroencephalogram, CSF: Cerebral spinal fluid,

+Progressive dementing illness accompanied by symptoms of memory impairment, executive dysfunction, disorientation, aphasia, visuospatial impairment

Table 6. Boxing data

| **Case no.** | **Age when started boxing** | **Duration of boxing (years)** | **Boxing career** | **Weight category** | **Estimated no. of fights** | **Official professional boxing record** | **No. of Knockout** | **Other history of head injury** | **Other potential exposure to RHI (Military service, sports & excessive alcohol consumption)** | **Other features which may be consistent with RHI** |
| --- | --- | --- | --- | --- | --- | --- | --- | --- | --- | --- |
| Case 1* | 11 | 14 | **Professional**  (He was a British and World Champion) | Bantamweight | 400 | W73-D3-L5(KO1) | 1  (According to official record) | He fractured his jaw in a brawl in his 50’s.  He had 3 RTA with head injury, severe scalp lacerations and right eye injury, he was treated as an in-patient after one of these RTA. | He served in the RAF for 6 months. | Cauliflower ears, broken nose, metacarpal exostoses of right hand. Ptosis and cataract of the right eye. Severe facial injury following loss of second fight in one contest. |
| Case 2*^ | 13 | 19 | **Professional**  (He started in booths then turned professional. He was a World Champion for some years) | Flyweight | 700 | W132-D1-L3(KO3) | 3  (According to official record) | RTA with LOC (aged 67) | - | - |
| Case 3* | 16 | 10 | **Professional**  (He won the Regional Championship) | Light-weight (He often fought as middleweight and sometimes heavyweight as ‘he could take it’) | 270 | W4-D3-L7(KO5) | 5  (According to official record) | Hit on the head by timber aged 26 with injury to 1^st^ cervical vertebral body | Heavy alcohol consumption with acute alcoholic pancreatitis aged 55 | - |
| Case 4 | 15 | 25 | **Professional**  (He was a challenger for a British Championship and boxed internationally. He had once fought a 15-round contest every day of the week with a 20- round contest on Saturday) | Lightweight | 600 | W25-D7-L31(KO7) | 7  (According to official record) | RTA with head injury with confusion and post-concussion symptoms when admitted in hospital. | He was an internationally footballer (soccer) as a boy.  He served in the Navy as a cook and was admitted in a hospital while serving in WWII, cause unknown.  Heavy alcohol consumption aged 35-40 at symptoms onset. | Visual impairment in late 20’s |
| Case 6* | 13 | 25 | **Professional**  (He mainly fought in booths competitions, ‘Mirror of Life’ Champion of England) | Feather-weight | 500 | W9-D3-L10(KO7) | 20  (Sometimes he took severe punishment to the head and could not always recall the fights that he had had the previous night) | Streetfights with known head injuries | - | - |
| Case 7* | 13 (According to official record) | 19 | **Professional** | Bantam-weight | 400 | W31-D3-L27(KO9) | 9  (According to official record) | RTA aged 57 | Excessive alcohol consumption since aged 50 at symptoms onset | Cauliflower ears, flattened nose |
| Case 8* | 17 | 23 | **Professional**  (He began as an amateur and soon turned professional. He had many contests, sometimes 3 per day and boxed throughout the world and was immensely successful) | NK | 565 | NK | NK | - | - | Cauliflower ears and broken nose with deviated septum |
| Case 9 | 19 | 12 | **Professional**  (He had a successful career, and was said to have fought at Blackfriars Ring and Madison Square Gardens) | NK | NK | NK | NK | - | Excessive alcohol consumption | Thickened left vocal cord from old haemorrhage from blow to throat |
| Case 10* | NK | NK | **Professional**  **(reputed)** | NK | NK | NK | NK | - | Excessive alcohol consumption | Loss an eye (unrelated to boxing) |
| Case 11 | NK | NK | **Professional**  **(reputed)** | NK | NK | NK | NK | - | He served in the Army and later the Maritime and were discharged from both on ‘health grounds’ | - |
| Case 12 | NK | NK | **Professional**  **(reputed)** | NK | NK | NK | NK | - | - | Registered blind (cause and diagnosis not known) |
| Case 13 | Teens | 30 | **Professional**  (He started boxing when he served in the Navy, won the heavyweight championship in Malta and several cups, took part in exhibition fights. He then boxed in the police force in his 20’s followed by teaching boxing in a local club and when he rejoined the Navy) | Heavy-weight | NK | NK | 2 or 3 | RTA aged 35 with head injury and LOC and post-concussion symptoms for up to a week.  Blast injury during WWII and he was ‘blown off a building’. | He served in the Navy for both WWI and WWII and served in the police force. | Cauliflower ears, broken his nose twice |
| Case 14 | 18 | 28 | **Amateur**  (He won the Wakefield RAF Boxing Trophy and a RAF Group Championship, had medals and a trophy) | Flyweight | NK | NA | 0 | - | He served in the RAF for 28 years. Played football (soccer). | - |
| Case 15 | NK | NK | **Amateur** | NK | NK | NA | 0 | Died of head injury in a RTA | He served in the Army. | - |

*: Cases with path-confirmed CTE applying the most recent NINDs criteria ([McKee, Cairns, et al., 2016](#_ENREF_17))

**^**This case (Case 2 in the Corsellis’ series ([Corsellis et al., 1973](#_ENREF_2))) corresponds to Case 3 reported in another historical boxer series ([Spillane, 1962](#_ENREF_29))

-: Absent, NK: Not known, W: Wins, D:Draws, L: Losses, KO: Knock outs, RTA: Road traffic accident, TIA: Transient ischaemic attack, LOC: Loss of consciousness, RHI: repetitive head injury, RAF: Royal Air Force, WWI: World War 1, WWII: World War 2,


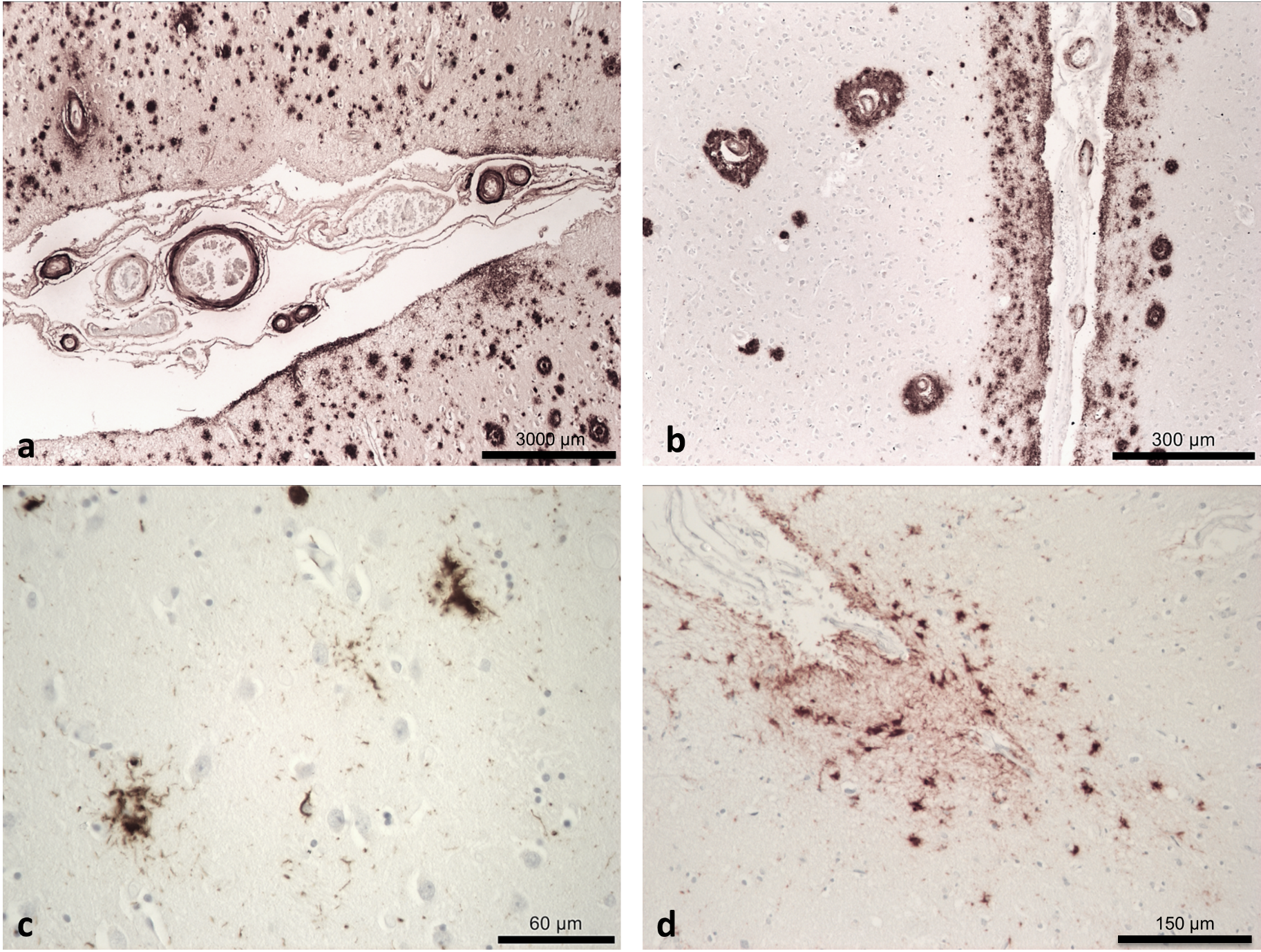


Figure 1 A) Aβ (4G8) immunohistochemistry. Aβ pathology within the frontal cortex with CAA in leptomeningeal vessels of case 10, age 67. B) Aβ immunohistochemistry. Aβ pathology within the occipital cortex revealing dyshoric vessel pathology, CAA and plaques. C) Tau (AT8) immunohistochemistry. High magnification image of tufted astrocytes and a coiled body in the caudate of case 4, age 68. D) Tau (AT8) immunohistochemistry. ARTAG tau deposition at the base of the sulcus of the frontal cortex in a similar pattern as CTE pathognomonic lesions (case 4).
